# The management of extremely preterm infants

**DOI:** 10.1186/1824-7288-40-S1-A9

**Published:** 2014-08-11

**Authors:** Fabio Mosca, Mariarosa Colnaghi, Monica Fumagalli

**Affiliations:** 1 NICU, Fondazione IRCCS Ca’ Granda - Ospedale Maggiore Policlinico, Università degli Studi di Milano, Milan, Italy

## 

Extreme prematurity is associated with an increased risk of mortality, morbidities and long-term neurodevelopmental impairment.

Optimizing prenatal, perinatal and postnatal care is essential to improve long-term outcome of extremely preterm babies.

Intrauterine growth restriction (IUGR) has been associated with a poorer neurological outcome and prenatal care should aim to an optimal balance between minimizing fetal injury or death versus the risks of iatrogenic preterm delivery. In the clinical phase of IUGR, hemodynamic adaptation occurs with blood flow redistribution preferentially to the brain, the so-called “*brain sparing effect*”. However, controversy remains as to whether “*brain-sparing*” indicates a higher risk of brain injury or is a protective mechanism [1].

The mechanisms underlying fetal growth restriction, also determine a limitation of lung growth and maturation, thus making the lung more vulnerable to postnatal damage and increasing the risk of bronchopulmonary dysplasia (BPD), the most significant respiratory complication of prematurity [2]. Current evidence suggests that the risk of BPD may be partially related to other antenatal factors as choriamniositis (CA), which is a leading cause of very preterm delivery. Whereas CA promotes lung maturation, mediated by prenatal inflammation mechanisms, and thereby decreases the severity of respiratory distress syndrome, it also seems to increase the risk of BPD, making the lung more susceptible to subsequent postnatal insults [3].

Neonatal resuscitation and postnatal management during the first minutes of life also play an important role in determining early and long-term outcome of VLBW. A multifaced intervention in respiratory care in the delivery room immediately after birth, including a sustained lung inflation procedure and a non invasive starting ventilation, seems to be effective in improving respiratory outcome [4].

Delayed umbilical cord clamping has received increasing attention in the management at birth of preterm infants as it seems to be beneficial and safe, being associated with less delivery room resuscitation interventions, improved haemodynamic stability and decreased rates of intraventricular hemorrhage [5].

Different pathophysiological mechanisms are involved in injuring the premature brain, in particular infection-inflammation, pre- and/or postnatal malnutrition, and abnormalities in systemic and cerebral haemodynamics and oxygen supply. Preventative measures are key to reduce neurological morbidities in an extremely preterm population and research projects are focusing on the possibility to stabilize cerebral oxygenation in the first days of life through the application of cerebral near-infrared spectroscopy oximetry and implementation of clinical treatment guidelines in order to reduce the risk of brain damage [6].
